# Suppressive Effects of Gelsemine on Anxiety-like Behaviors Induced by Chronic Unpredictable Mild Stress in Mice

**DOI:** 10.3390/brainsci12020191

**Published:** 2022-01-30

**Authors:** Hui Yu, Mo-Huan Tang, Zi-Yue Zeng, Si-Juan Huang, Xiao-Feng Zheng, Zhao-Ying Liu

**Affiliations:** 1 College of Veterinary Medicine, Hunan Agricultural University, Changsha 410128, China; 17605032171@sina.cn (H.Y.); tangmohuan@sina.cn (M.-H.T.); 19973881087@sina.cn (Z.-Y.Z.); 13657322320@163.com (S.-J.H.); xiaofengzheng125@hotmail.com (X.-F.Z.); 2 Hunan Engineering Technology Research Center of Veterinary Drugs, Hunan Agricultural University, Changsha 410128, China

**Keywords:** gelsemine, anxiety, BDNF, NLRP3 inflammasome, *Gelsemium*

## Abstract

Gelsemine is an active principle and a major alkaloid found in *Gelsemium* genus of plants belonging to the *Loganiaceae* family. The aim of the present study was to explore whether gelsemine exerts anxiolytic effects on a mouse model of chronic-unpredictable-mild-stress (CUMS)-induced anxiety-like behaviors. NOD-like receptor protein 3 (NLRP3) inflammasome, downregulated cAMP-response element-binding protein (CREB) and brain-derived neurotrophic factor (BDNF) were also evaluated as potential mechanisms. First, gelsemine reversed a CUMS-induced decrease in body-weight gain in mice. Next, gelsemine alleviated CUMS-induced anxiety-like behaviors, as evidenced by the increased distance traveled in the central zone of the open-field test, both the increased percentage of time spent and distance traveled in the light compartment, the increased number of transitions between compartments in the light/dark-transition test, and the increased percentage of entries and time spent in the open arm of the elevated plus-maze. In addition, gelsemine decreased the levels of pro-inflammatory cytokines, including interleukin (IL)-1β and IL-6, in the hypothalamus and hippocampus of CUMS mice. Interestingly, further investigations revealed that gelsemine inhibited the CUMS-induced activation of NLRP3-inflammasome pathways and downregulated CREB and BDNF overexpression in the hypothalamus. In summary, gelsemine alleviated anxiety-like behaviors in the CUMS-induced mouse model. Gelsemine exerted its anxiolytic effects by modulating the NLRP3 and CREB/BDNF pathways.

## 1. Introduction

Anxiety disorders are the most common psychiatric illnesses among children and adolescents, as 10–20% of individuals have experienced this disorder during their lifetime [1]. When left untreated, anxiety symptoms persist and can result in intense disabling consequences associated with a disturbance in the quality of life, an increased incidence of unemployment, and a substantial personal and socioeconomic burden [1]. Currently, seven anxiety syndromes have been identified, including panic disorder, agoraphobia, social anxiety disorder, generalized anxiety disorder, specific phobias, separation anxiety disorder, and selective mutism [2,3]. The pathogenesis of anxiety disorders involves neurobiochemistry [4,5], neuroendocrine signaling [6], the immune system [7] and molecular genetics [3,8]. Drug therapy is a typical first-line treatment, and the effect of drug treatment is greater than psychological interventions [9]. Currently, the anti-anxiety drugs used in the clinic mainly include antidepressant anxiolytics, benzodiazepine anxiolytics, nonbenzodiazepine anxiolytics, and anticonvulsant anxiolytic drugs [10]. Although many pharmacological treatment options are available, many anti-anxiety medications used to treat anxiety have negative side effects, including addiction, depression, suicide, seizures, sexual dysfunction, and headaches, among others [10]. Consequently, novel anti-anxiety drugs with better tolerance and lower side effects must be developed.The development of novel therapies for the treatment of anxiety disorders from natural resources inspired by traditional medicine has attracted increasing attention, which is one of the important directions of new drug research and development [1,11].

Based on accumulating evidence, neuroinflammation contributes to the development of anxiety [12,13]. According to several clinical studies, the levels of pro-inflammatory cytokines, such as IL-1β and IL-6, are significantly increased in the brains of patients with anxiety and in animal models [12]. These excess pro-inflammatory cytokines might result in cellular damage, impaired neural plasticity, and the suppression of neurogenesis [2]. The NOD-like-receptor-protein-3 (NLRP3)-inflammasome pathways contribute to the modulation of pro-inflammatory cytokines [2,14,15,16]. Thus, molecules that inhibit NLRP3 pathways and reduce the levels of pro-inflammatory cytokines may be a promising strategy for the development of anti-anxiety drugs.

Normally, neurotrophic factors mediate neuronal survival and differentiation [14]. Brain-derived neurotrophic factor (BDNF) is one of the most well-studied neurotrophins in the healthy and diseased brain [17,18]. Alterations in BDNF expression may affect anxiety-related behaviors [17]. Furthermore, inhibitors that block overexpressed BDNF have been shown to reduce anxiety [19]. Moreover, the cAMP-response element-binding protein (CREB) is a well-reported phosphorylation-dependent transcription factor [14,20]. CREB regulates the expression of genes closely associated with neuronal survival and differentiation, neurogenesis, and synaptic plasticity [21]. An abnormal CREB level was recently implicated in pathological conditions of anxiety [22]. CREB functions as an upstream transcription factor of BDNF that regulates the transcription and subsequent expression of BDNF [14]. Therefore, CREB and BDNF pathways are also related to anxiety disorders.

*Gelsemium*, a small genus of the family *Loganiaceae* [23,24], comprises three popularly known species. The first is a yellow jasmine known as *Gelsemium sempervirens* Ait in the southwestern United States [25], which has traditionally been used to treat a variety of anxiety, pain and other diseases, as well as for homeopathy at low doses or even ultralow doses [26,27].The second is the rarely reported North American *Gelsemium rankinii* Small [28]. The last is *G. elegans* (*Gelsemium elegans*) distributed in Asia. *G. elegans* is the earliest member of this family recorded in China; it is also called “Gou Wen” or Heartbreak grass. *Gelsemium* is also generally known as a highly toxic plant.There have been numerous reports of poisoning, and the typical symptoms include chest tightness, convulsions, continuous respiratory irregularities, arrhythmias, among which respiratory failure and respiratory arrest are the main causes of death [28].However, in traditional pharmacology, it possesses a variety of pharmacological activities, such as an analgesic effect [29], anti-stress activity [30], and anti-anxiety activity [31].

In fact, very low doses of *Gelsemium* extracts reduce anxiety in animal models [31]. The alkaloids gelsemine, koumine, gelsenicine, and gelsevirine constitute the primary active molecules of *G. elegans*. More recently, some articles have reported that gelsemine reduces stress-induced behavioral alterations in mice in the elevated plus-maze (EPM),open-field test (OFT) and light/dark-transition (LDT) test [11,32]. Currently, the general hypothesis about the anti-anxiety mechanism of gelsemine is that it may be related to the regulation of the levels and activities of neurotransmitters such as γ-aminobutyric acid and allopregnenolone in the spinal cord and their receptors [32,33]. However, none of the current research on the anxiolytic effect of gelsemine has established a stable anxiety model resembling the clinicopathological characteristics. Chronic stress has been linked to the pathophysiology of various psychiatric disorders, including anxiety disorders and depression [34]. Researchers have all been directly subjecting normal animals to behavioral tests and observing the anti-anxiety effect of gelsemine under stress conditions. The anti-anxiety actions and underlying mechanisms in a mouse model of anxiety have not been precisely elucidated.

Therefore, in this paper, the mouse model of anxiety induced by chronic unpredictable mild stress (CUMS) was established as previously described, with minor modifications [35] to evaluate the effects of gelsemine on anxiety in CUMS-induced mice for the first time. NLRP3/IL-1β/IL-6 and BDNF/CREB pathways were also evaluated as potential mechanisms.

## 2. Materials and Methods

### 2.1. Animals

Male ICR mice (6–8-week-old, 18–22 g) were purchased from Hunan SJA Laboratory Animal Co. Ltd. (Certificate number SCXK 2016-0002, Changsha, China). For breeding and maintenance, mice were group housed (maximum of five animals per cage) with free access to food and water. Animals were housed under standard specific pathogen-free conditions at a temperature of 24 ± 2 °C and humidity of 50 ± 15% on a 12 h light/dark cycle (lights on 8 a.m. to 8 p.m.). The mice were acclimated to the laboratory environment for 5 days before the experiment. All animal experiments were conducted according to the Nation Institutes of Health Guide for the Care and Use of Laboratory Animals, and the animal protocol was approved by Hunan Provincial Laboratory Animal Center of Hunan Center for safety Evaluation and Research of Drugs (IACUC-2019(3)024, Changsha, China).

### 2.2. Mouse Model of CUMS

**Figure 1:**
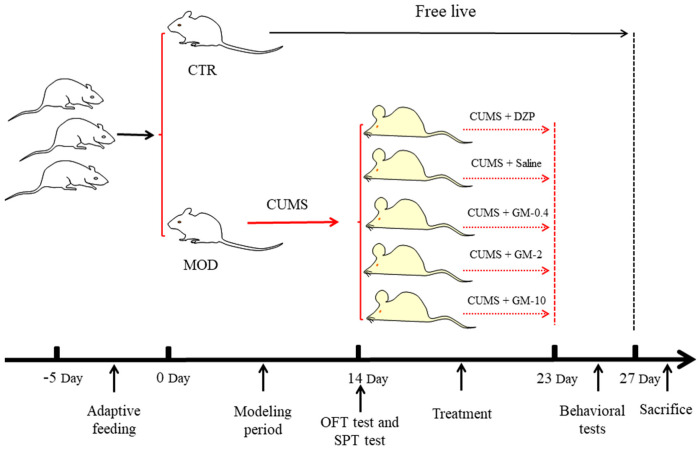
Scheme of the experiments used to assess the effects of gelsemine on anxiety-like behaviors in mice. Seven-week-old mice were randomized into two groups (CTR = 12; CUMS = 60). The mice in which the anxiety model was successfully constructed were randomly divided into 5 groups (*n* = 12 mice/group). (gelsemine/GM: 0.4 mg/kg, 2 mg/kg, or 10 mg/kg; diazepam/DZP: 1 mg/kg).

Seventy-two mice were randomly divided into two groups, including the control (CTR; *n* = 12) and CUMS-model (CUMS; *n* = 60) groups. For each experiment, 72 mice were randomly distributed, four per cage, in plastic cages (size: 30 × 14 × 12 cm) and housed with food and water available ad libitum, except during the brief testing periods and the modeling period. Mice in the control group were housed under standard laboratory conditions on a 12 h light/dark cycle with food and water available ad libitum. Mice in the CUMS group were housed in a separate room. The procedure for establishing the CUMS group was performed as described in previous reports, with slight modifications [14,34]. The animals were subjected to CUMS daily. Protocols were randomly scheduled and changed daily to ensure that the procedure remained unpredictable to the animals. The stressors included (1) forced swimming for 10 min, (2) stroboscopy (12 h), (3) withholding water (12 h), (4) fasting (12 h), (5) switching the day and night cycle (illumination overnight, 12 h), (6) cage tilting (12 h), (7) forced physical restraint (12 h), and (8) a wet cage (12 h).On day 14, the CUMS-model group was further randomized into five groups (*n* = 12), including the model group (MOD), CUMS + gelsemine (GM-0.4, 2, 10 mg/kg) and CUMS + diazepam (DZP, 1 mg/kg). The doses of diazepam (1 mg/kg) and gelsemine (GM-0.4, 2, 10 mg/kg) were selected based on the anxiolytic and antidepressant activity of these agents in previous reports [11]. The gelsemine was purchased from Chengdu Munster Biotechnology Co., Ltd. (Chengdu, China). All drug solutions were freshly prepared on the test days and administered intraperitoneally at a volume of 0.2 mL/10 g. The conditions used to induce CUMS were performed for 23 days, and gelsemine, saline or DZP was administered daily at 9:00 am by intraperitoneal injection from the 14th day (including CTR group) and lasting for 9 days. The timeline of the experimental treatment is shown in Figure 1.

### 2.3. Behavioral Tests

In order to verify whether the animal-anxiety model was successful, the sucrose-preference test (SPT) and open-field test (OFT) were performed on the 14th day. The subsequent experiments were performed in the following order: the open-field test (OFT) was on the 23rd day, the light/dark-transition (LDT) test was on the following day, the elevated plus-maze (EPM) was on the 25th day, and the forced-swim test (FST) was on the 26th day. Just before testing, the animals were allowed to acclimate to the room inside their cages for 3 min after being moved from their customary housing area. The operators stayed outside the testing room during recording of the experimental sessions.

#### 2.3.1. Sucrose-Preference Test (SPT)

The sucrose-preference test was performed using a previously described method, with slight modifications [36].Briefly, twenty mice were randomly selected from the control group (*n* = 10) and the CUMS group (*n* = 10) on the 14th day. The mice were not provided food or water for 12 h before the experiment. The mice were then given a free choice of two bottles containing a 1% sucrose solution (*w*/*v*) for 12 h. The two bottles were replaced with one bottle containing the 1% sucrose solution (*w*/*v*) and the other containing sterile water. After 12 h, the volumes of water and sucrose solution consumed were measured, and the sucrose-preference coefficient was calculated using the following formula:

(1) $$\mathrm{Sucrose}\;\mathrm{preference}=\frac{\mathrm{Sucrose}\;\mathrm{consumption}}{\mathrm{Water}\;\mathrm{consumption}\;+\;\mathrm{sucrose}\;\mathrm{consumption}}\times 100\%$$

#### 2.3.2. Open-Field Test (OFT)

An open-field test [37,38] was conducted using black boxes (25 × 25 × 31 cm) equipped with white-light illumination (100 lx) to determine the effects of gelsemine on spontaneous locomotor activities. The center of the field reflects the exploratory tendency of the animal. However, the edge of the field reflects anxiety and the desire of the animal to escape. The mice were placed at the edge of the field and allowed to adapt to the field environment for 5 min. The spontaneous locomotor activities (total distance traveled, the distance traveled in the center, and the distance traveled along the edge) of each mouse were recorded for 5 min using a video-tracking system (DigBehv, Jiliang Software Technology Co., Ltd., Shanghai, China). The test box was thoroughly cleaned with 75% ethanol between each test. 

#### 2.3.3. Light/Dark-Transition (LDT) Test

The LDT test [39,40] is widely used to screen anxiolytic drugs in rodents and is based on their increased aversion to brightly illuminated areas in response to stressors [11]. In the test, the inquiry behavior of the mouse is suppressed by illumination. The administration of anxiolytic will increase the number of times the mouse passes through the light and dark compartments and the residence time in the bright compartment of the apparatus.

The test apparatus consisted of a secure dark compartment (15 × 15 cm) and an illuminated compartment (15 × 15 cm). The two compartments were connected by an open hole (4 × 4 cm), which allowed the animals to pass from one compartment to the other. Each mouse was placed in the center of the illuminated compartment. Animal behavior was recorded using a camera for 5 min. The time spent in the light compartment and the number of transitions between the light and dark compartments were examined using video-analysis software. After each test, both compartments were wiped with 75% ethanol to remove potential interference from odors.

#### 2.3.4. Elevated Plus-Maze (EPM)

The EPM is a classic test used to examine the anxiety state of the test subject and has been validated for measuring anxiolytic-like activities in rodents [41,42]. The apparatus consisted of two opposite open arms (16 × 5 cm) and two enclosed arms (16 × 5 × 12 cm) extending from a common central platform (5 × 5 cm). The maze was placed 45 cm above the floor. Mice were individually placed at the center of the apparatus facing an open arm. The time spent in each arm and the number of entries into each arm was recorded for 5 min using video-analysis software. After each test, the maze was wiped with 75% ethanol to remove interference from odors.

An entry was defined as all four paws having crossed the line between an arm and the central area.Both the percentage of time spent and the number of entries into open arms (open entries/total entries × 100; open time/total time × 100) were recorded as indicators of anxiety-like behaviors. The percentage of time spent in the open arms and the percentage of open-arm entries and closed-arm entries were used as measures of anxiety.

#### 2.3.5. Forced-Swim Test (FST)

The FST is the most widely used in vivo test to assess antidepressant activity [43]. Mice were individually placed in a glass cylindrical aquarium (10 × 25 cm) containing 15 cm of water (24 ± 2 °C) for 5 min. The time spent immobile was manually recorded for 5 min. Immobility was defined as the time mice spent making only the movements necessary to keep their heads above water. The water was changed after each test.

### 2.4. Brian-Tissue Collection

After the behavioral tests, animals were decapitated and then brain tissues were collected. Afterwards, brains were harvested and the prefrontal cortex, the corpus striatum, hippocampus and hypothalamus were dissected on ice and rapidly frozen with liquid nitrogen. All samples were stored at −80 °C until the assay.

### 2.5. Measurement of Inflammatory Cytokine Levels 

The concentrations of pro-inflammatory cytokines (IL-6 and IL-1β) in brain tissues were measured using ELISA kits (Elabscience, Wuhan, China). Briefly, a brain homogenate (10%) was prepared by homogenizing the brain tissue in normal saline, and supernatants were harvested and analyzed using ELISA according to the manufacturer’s protocol. All results were then normalized to the total protein concentration measured with a BCA kit.

### 2.6. Histopathology

The collected brains tissues were immediately fixed with 4% paraformaldehyde. Then, the fixed tissues were dehydrated through a graded series of ethanol, hyalinized with xylene, embedded in paraffin, and 5 µm sections were cut and placed on glass slides. The sections were stained with hematoxylin and eosin (HE) for histological assessment. Digital images were obtained using a Nikon Eclipse Ci-L microscope at a fixed 100× magnification.

### 2.7. Western Blot Analysis

The tissues were washed with pre-cooled PBS to remove blood stains. Then, protease inhibitors and lysis buffer were added for homogenization on ice. Total proteins were extracted from the hippocampus and hypothalamus in 150 μL of RIPA buffer (Servicebio, Wuhan, China) containing 1 mM PMSF (Servicebio, Wuhan, China). Protein concentrations were determined using the BCA Protein Assay Kit (Beyotime, BioTECH, Haimen, China) according to the manufacturer’s instructions. Approximately 30 g of total protein were separated by sodium dodecyl sulfate-polyacrylamide gel electrophoresis (SDS-PAGE) and transferred to a polyvinylidene fluoride (PVDF) membrane (Millipore, 0.22 μm, 0.45 μm, Millipore, Germany). Membranes were blocked with 5% fat-free milk in TBST (Tris HCl, NaCl and Tween 20) for 1 h at room temperature, and then incubated overnight at 4 °C with the following primary antibodies: BDNF (rabbit polyclonal antibody1:1000, ABclonal, Wuhan, China), CREB (rabbit polyclonal antibody, 1:1000, ABclonal, Wuhan, China), NLRP3 ((rabbit polyclonal antibody, 1:500, ABclonal, Wuhan, China), and β-actin (1:5000, Servicebio, Wuhan, China). Membranes were rinsed with TBST five times for 5 min each and incubated with the following secondary antibodies for 1 h at room temperature: horseradish-peroxidase (HRP)-conjugated Affinipure Goat Anti-Mouse IgG (H + L) (1:5000, Servicebio, Wuhan, China) or HRP-conjugated Affinipure Goat Anti-Rabbit IgG (H + L) (1:5000, Servicebio, Wuhan, China). Membranes were then rinsed as described above, and treated with ECL (Servicebio, Wuhan, China), which was detected using a chemiluminescence detector (Bio-Rad, Hercules, CA, USA). The level of each protein was measured with Image Lab software.

### 2.8. Data Analysis

The data were analyzed using GraphPad Prism software (GraphPad Software Inc., San Diego, CA, USA). In this experiment, statistical analysis of the differences between two groups was performed using one-way ANOVA with Bonferroni’s correction. All data are represented as means ± S.E.M. with *p* values < 0.05 being considered statistically significant.

## 3. Results

### 3.1. Establishment of the Mouse Model of CUMS-Induced Anxiety

**Figure 2:**
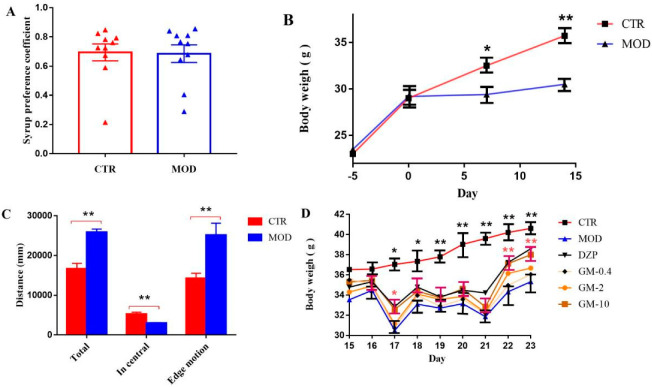
Establishment of the mouse model of CUMS-induced anxiety-like behaviors and decrease in body-weight gain. (**A**) Twenty mice were selected from the control group (*n* = 10) and the CUMS group (*n* = 10) on the 14th day and were subjected to the sucrose-preference test. (**B**) The body weights of mice in the control group and the CUMS group were recorded 5 days before experiment and 0, 7 and 14 days during the experiment. (**C**) Twenty mice were selected from the control group (*n* = 10) and the CUMS group (*n* = 10) on the 14th day and were tested in the open-field apparatus. (**D**) The body weights of mice in the six groups were recorded for 9 days. * *p* < 0.05, ** *p* < 0.01, compared with the model group.

A mouse model of anxiety induced by CUMS was constructed to ensure that our results would closely resemble the clinicopathological features of anxiety disorders. Ten mice each from the control group and the CUMS-model group were randomly selected to perform the SPT and OFT in order to ensure the reliability of subsequent experiments and the successful establishment of the model. After validation, 20 mice were returned to the original group for subsequent experiments.The results show that no difference was observed in the sucrose-preference coefficient between the control group and the CUMS-model group, indicating that the model group did not exhibit depression (Figure 2A, *p* = 0.92, versus the control group). As shown in Figure 2B,D (*p* < 0.01, versus the control group), the mice were anxious after physical stimulation, and the body weight of the CUMS group was significantly decreased after the 7th day and persisted until the 23rd day compared with the control group. As shown in Figure 2C, the open-field experiment showed a significantly greater total distance traveled in the open field and distance traveled on the edge by the model group than by the control group (*p* < 0.01, versus the control group), while the distance traveled in the central area was significantly lower than control group (*p* < 0.01; versus the control group). Based on the above experimental results, the mouse-anxiety model we constructed was successful. We recorded changes in the weights of mice during treatment to determine whether gelsemine reversed the CUMS-induced decrease in body weight. As illustrated in Figure 2D, the effect of DZP (*p* < 0.01, versus the model group) was the same as that of gelsemine (*p* < 0.01, versus the model group), whichreversed the CUMS-induced decrease inbody weight.

### 3.2. Effects of Gelsemine on Anxiety-like Behaviors in Mice

#### 3.2.1. OFT

**Figure 3:**
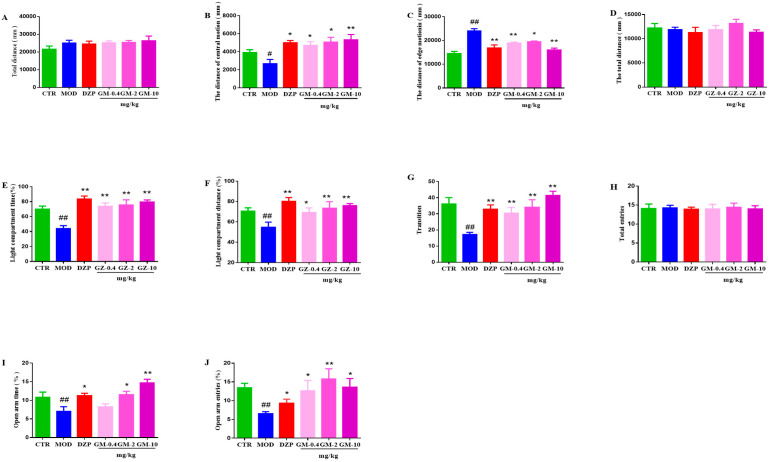
The anxiety-like effects of gelsemine on mice exposed to chronic unpredictable mild stress (CUMS). Mice were exposed to stress (subjected to CUMS) and treated daily with saline, gelsemine (GM, 0.4, 2.0 and 10.0 mg/kg, i.p.), diazepam (DZP, 1.0 mg/kg, i.p.) before testing for 9 days. The total distance, the distance traveled in the central area and the distance traveled along the edge were measured in the OFT on day 24 ((**A**–**C**), respectively). The total distance traveled, the percentage of time spent in the light compartment, the percentage of distance traveled in the light compartment and the number of transitions between compartments were measured in the LDT on day 25 ((**D**–**G**), respectively). The closed-arm entries, percentage of time spent in the open arms and percentage of open-arm entries were measured in the EPM on day 26 ((**H**–**J**), respectively). Data are presented as means the ± SEM (*n* = 12). # *p*< 0.05, ## *p* < 0.01, compared with the control group, * *p* < 0.05, ** *p* < 0.01, compared with the model group.

As shown in Figure 3A–C, the total distance traveled in the OFT by mice in all groups was not affected (*p* = 0.96, versus the control group). However, the CUMS group exhibited a significant decrease in the distance traveled in the central area (*p* < 0.01 compared with the control group), and a significant increase in distance traveled along the edge (*p* < 0.01 compared with the control group). After treatment with gelsemine (0.4, 2 or 10 mg/kg) or DZP (1 mg/kg), the CUMS mice significantly reversed the behavioral alterations compared to the model group (Figure 3B,C).

#### 3.2.2. LDT

As shown in Figure 3D–G, CUMS exposure significantly decreased the percentage of the time spent (*p <* 0.01, versus the control group) and the distance traveled (*p <* 0.01, versus the control group) in the light compartment, as well as the number of transitions between compartments in the LDT by the stressed-vehicle mice compared with the control-vehicle animals (*p <* 0.01, versus the control group). Gelsemine (0.4, 2 or 10 mg/kg) treatment induced a noticeable increase in the percentage of time spent and distance traveled in the light compartment, and an increase in the number of transitions between compartments in CUMS mice compared to vehicle-treated CUMS-exposed mice (*p <* 0.01 compared with the model group). Similar results were obtained after DZP (1 mg/kg) administration (*p <* 0.01 compared with the model group).

#### 3.2.3. EPM

Figure 3H–J illustrates the effect of gelsemine (0.4, 2 or 10 mg/kg) on the percentage of entries and time spent in the open arm and closed-arm entries in the EPM. The statistical analysis revealed that chronic stress significantly increased anxiety in mice, as indicated by the reduced percentage of open-arm entries (*p <* 0.01, versus the control group) andtime spent in the open arms (*p* < 0.01, versus the control group) and increases in the closed-arm entries (*p <* 0.01, versus the control group). After treatment with gelsemine (0.4, 2 or 10 mg/kg) or DZP (1 mg/kg), increases in the percentage of open-arm entries and time spentin the open arms, and reductions in the closed-arm entries were observed compared with the model animals. However, statistically significant differences in total arm entries were not observed between groups.

#### 3.2.4. FST

As shown in Supplementary Materials Figure S1A,B, no significant differences in swimming and climbing time (*p* = 0.97, versus the control groupand immobility time (*p* = 0.85, versus the control group) were observed between groups in the compulsive-swimming experiment.

### 3.3. Gelsemine Decreased Inflammation in the Mouse Brain

**Figure 4:**
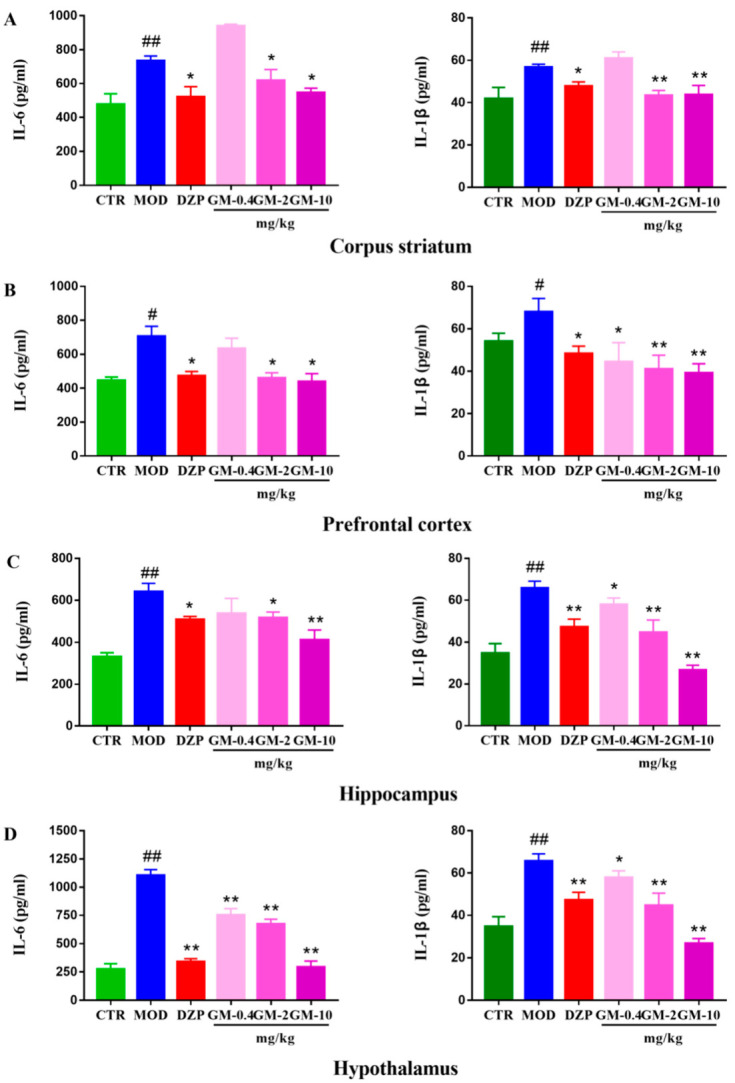
Effects of gelsemine on inflammatory cytokines IL-6/IL-Iβ expression in corpus striatum (**A**), prefrontal cortex (**B**), hippocampus (**C**) and hypothalamus (**D**). Data are presented as means the ± SEM (*n* = 5). # *p* < 0.05, ## *p* < 0.01, compared with the control group, * *p* < 0.05, ** *p* < 0.01, compared with the model group.

CUMS significantly increased the levels of IL-6 and IL-1β (Figure 4) in the hypothalamus (Double *p* values *<* 0.01, versus the control group), prefrontal cortex (*p <* 0.05; *p* < 0.01, versus the control group), striatum (Double *p* values <0.01, versus the control group) and hippocampus (*p <* 0.01, versus the control group) compared with the control group. Notably, the effect of CUMS was more pronounced on inflammation in the hypothalamus than in the other regions. Gelsemine (2.0–10 mg/kg) and DZP (1 mg/kg) treatments markedly decreased the CUMS-induced increase in the IL-6 and IL-1β levels compared to the CUMS group. 

### 3.4. Gelsemine Ameliorates Changes in the Brains of Anxious Mice, as Observed Using Electron Microscopy

**Figure 5:**
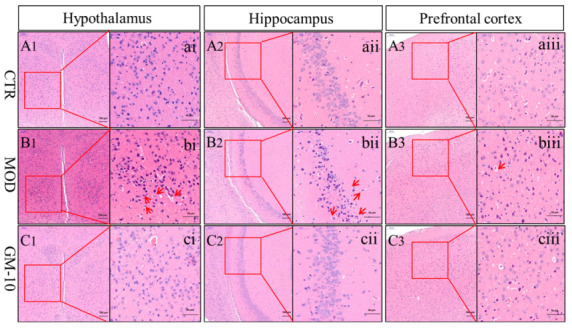
Representative images of HE staining in mice hypothalamus, hippocampus and prefrontal-cortex regions. (**A1**–**A3**) represent the control group (×100), (**B1**–**B3**) represent the model group (×100), (**C1**–**C3**) represent the 10 mg/kg gelsemine-treated group (×100); other image represents the same group with ×400 magnification. The arrows in the model group indicate damaged cells.

Tissue sections were stained with HE to evaluate the histopathological characteristics of the brains of anxious mice and the effect of gelsemine on the brain tissue. As shown in Figure 5, the cell structure of damaged neurons in the hypothalamus and hippocampus of the mice exposed to CUMS was blurred, and a small amount of fragmentation occurred. In contrast, the gelsemine treatment reduced the extent of damage in anxious mice and ameliorated CUMS-induced changes in these brain regions. However, no changes in the corpus striatum were observed among the control group, the model group, and the gelsemine-treatment group (Supplementary Materials Figure S2). 

### 3.5. Gelsemine Inhibits the CUMS-Induced Increase in the Expression of CREB, BDNF and NLRP3 Inflammasomes

**Figure 6:**
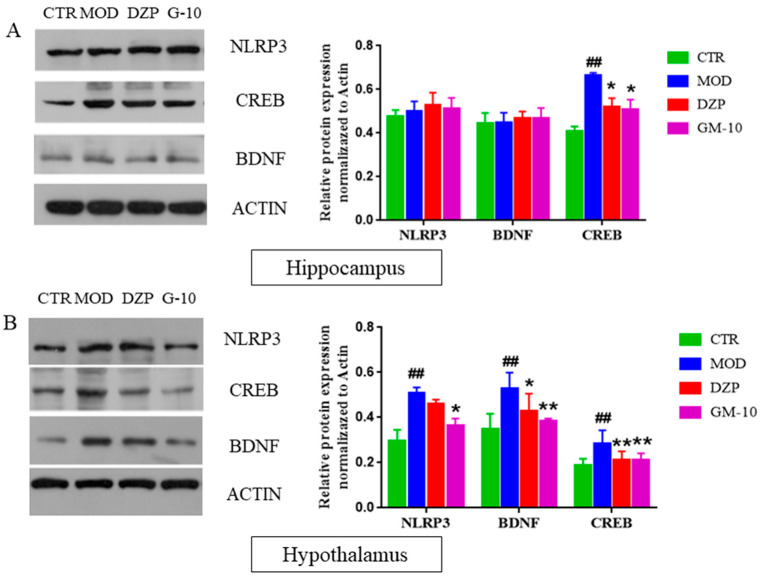
Gelsemine inhibits CREB, BDNF and NLRP3 inflammasomes in the hippocampus (**A**) and hypothalamus (**B**) of CUMS-induced mice. The quantitative analysis of CREB, BDNF and NLRP3 inflammasomes after gelsemine treatment of CUMS-induced mice. Data are presented as means the ± SEM (*n* = 5) in three independent experiments. ## *p* < 0.01, compared with the control group, * *p* < 0.05, ** *p* < 0.01, compared with the model group.

The expression of CREB and BDNF in different regions of the mouse brain was determined in order to analyze the effects of gelsemine on the CREB/BDNF and NLRP3 pathways. As shown in Figure 6, CUMS stimulation increased the expression of BDNF, CREB and NLRP3 inflammasomes in the hypothalamus (Figure 6B, *p <* 0.01; *p* < 0.01; *p* < 0.01, versus the control group) and only increased CREB expression in the hippocampus (Figure 6A, *p <* 0.01).However, gelseminetreatment dramatically reversed the changes in CREB and BDNF expression. However, gelsemine treatment dramatically decreased the levels of CREB, BDNF and NLRP3 inflammasomes in the hypothalamus of CUMS-stimulated mice.

## 4. Discussion

The comorbidity of anxiety and depression is quite common in clinical practice [3,8]. In the animal model of anxiety, most animals show both an anxiety response and depression response. Upon short-term exposure to stress, the animals mainly present the anxiety response, while chronic stress significantly increases the behavioral changes associated with depression [44]. In general, a CUMS-induced depression model requires sustained stimulation over a long period of time, typically approximately 5–6 weeks [14]. Some reports have suggested that there are differences in responsiveness to CUMS according to the different strains of mice and behavioral tests [45,46]. Jung et al. demonstrated that ICR mice are appropriate for evaluating stress-induced anxiety-like behaviors in SPT and OFT [45]. Therefore, ICR mice were chosen for the anxiety model for the CUMS induced in this paper. In contrast to previous reports and research methods, our paper is the first to construct an animal model of anxiety for evaluating the effects of gelsemine on anxiety. In this paper, ICR mice were placed in a series of stressful situations. Following short-term stimulation for 14 days, the mice developed anxiety disorders resembling the clinicopathological characteristics. In the current study, stimulation with CUMS managed to create an anxiety phenotype in mice, as evidenced by the significant decrease in the distance traveled in the central area and the increase in the distance traveled along the edge in the OFT. Moreover, the body weight of the CUMS group was significantly decreased. Then, the gelsemine treatment reversed the decrease in the body weight and the behavioral alterations observed in the CUMS-treated mice.

Behavioral research provides clues to the study of human anxiety, and thus the study of anxiety disorders requires the support of animal models. The open-field test is used to analyze locomotion, anxiety and stereotypical behaviors. Changes in locomotion may indicate changes in neural processes that potentially reflect abnormalities in brain function [47]. In the OFT, gelsemine produced the same significant anxiolytic effects as diazepam, significantly increasing the distance traveled in the central area of the field and reducing the distance traveled in the marginal area of the field. As reported in previous articles, the animal in an anxiety state tends to enter arms faster, decrease the residence time in the light compartment or reduce the number of transitions between compartments in the LDT test [26]. Gelsemine significantly increased the residence time and percentage of entries in the light compartment by the mice in a dose-dependent manner, while the number of transitions between compartments was also significantly increased. Compared with the standard anti-anxiety drug DZP, the anti-anxiety-like effect of gelsemineappeared to be better. 

The EPM plays an important role in screening anti-anxiety drugs and determining the biochemical mechanism of anxiety. Theoretically, mice with moderate anxiety will enter the open arms of the maze more frequently, while more anxious mice tend to spend more time in the closed arms [48]. As reported in previous articles, *G. elegans* alkaloids exert a good anti-anxiety-like effect [49]. Based on our experimental results, similar to the anti-anxiety effect of DZP, gelsemine increased the percentage of open-arm entries and the time mice spent in the open arms, while it decreased the time mice spent in the closed arms in a dose-dependent manner. In addition, in the FST, no significant differences in the suspension time and the time spent swimming and struggling were observed between each group of mice. Abnormal locomotion might affect performance in the OFT, LDT and EPM. However, the doses of gelsemine used did not disrupt spontaneous motor activity. Significant differences in the total distance traveled in the OFT, LDT and EPM were not observed between groups. Thus, the present results, together with findings from other studies, indicate that gelsemine has the potential to be developed as an anti-anxiety drug in the future.

Based on accumulating evidence, anxiety is accompanied by inflammation in the CNS, and overproduction of pro-inflammatory cytokines plays an important role in the pathophysiology of anxiety [50]. Normally, low levels of pro-inflammatory cytokines are detected in the central nervous system and regulate neuronal survival and function [14]. After inflammatory signaling (e.g., stress and pro-inflammatory signals), high levels of pro-inflammatory cytokines generate a sequence of events that may eventually lead to neuronal dysfunction and apoptosis [17]. Consistent with previous reports, gelsemine inhibited neuroinflammation and reduced the expressions of inflammatory cytokines [51,52]. In the current study, we revealed the inhibitory effect of gelsemine on CUMS-induced increases in IL-1β and IL-6 levels. Furthermore, the inhibitory effect on inflammatory-cytokine production was also reported for other anxiolytics, such as gastrodin, honokiol and mangiferin [1]. The activation of the NLRP3 inflammasome is another common regulator of inflammatory processes [14]. Active caspase-1 and mature IL-1β are then released into the extracellular environment and contribute to inflammatory and apoptotic processes [14]. As shown in the current study, gelsemine suppressed the activation of the NLRP3 inflammasome. Consistently, the gelsemine-mediated inhibition of the activity of the NLRP3 inflammasome was confirmed in the current study, which directly supported the inhibitory effect of gelsemine on CUMS-induced neuroinflammatory responses.

The level of CREB in the brain regulates anxiety symptoms to some extent, and the level of CREB expression also reflects the severity of anxiety symptoms to some extent [53]. CREB is strongly associated with BDNF expression; it is an upstream transcription factor of BDNF, and the expression of BDNF mainly depends on CREB activation [14]. BDNF plays an important role in modifying the brain in response to stress-inducing stimuli [54]. It is involved in anxiety-like behaviors in preclinical models, is involved in the mechanism regulating synaptic plasticity and the formation of neural circuits and is a key mediator of synaptic plasticity in the fear circuit [55]. BDNF not only promotes the differentiation of dopaminergic, serotonergic, and GABAergic neurons but also protects nerves, meta and glial cells from oxidative stress [55]. Following stimulation, the level of inflammation in the mouse brain is increased, which will damage neurons in the brain and lead to abnormal brain function. In the experiment, the gelsemine-mediated inhibition of the expression levels of CREB and BDNF in the hypothalamus might be due to decreased levels of IL-1β and IL-6, and thus requires further study.

## 5. Conclusions

In summary: gelsemine may ameliorate stress-related anxiety-like behaviors in mice. The mechanism underlying the anti-anxiety-like effect of gelsemine may be to protect neurons by suppressing the occurrence and development of inflammation by modulating the NLRP3 and CREB/BDNF pathways. Therefore, gelsemine has the potential to be developed as an anti-anxiety drug in the future.

## Data Availability

The data presented in this study are available in the article.

## Supplementary Materials

The following are available online at https://www.mdpi.com/article/10.3390/brainsci12020191/s1, Figure S1: The immobility time and swimming and climbing time were measured in FST on day 27, Figure S2: Effect of Gelsemine on the pathomorphology of Corpus striatum in mice.

## Abbreviation

CNS	Centra + B17 + A1:B17 + A1:B16 + A1:B17
*G. elegans*	*Gelsemium elegans*
GM	Gelsemine
DZP	Diazepam
CUMS	Chronic unpredictable mild stress
MOD	Model group
CTR	Control group
CREB	cAMP-response element-binding protein
BDNF	Brain derived neurotrophic factor
SPT	Sucrose-preference test
OFT	open-field test
EPM	Elevated-plus-maze test
LDT	light/dark-choice test
FST	Forced-swim test
IL-6	Interleukin 6
IL-1β	Interleukin 1 Beta
NLRP-3	NOD-like receptor protein 3
