# Supplementary material for: Suppressive Effects of Gelsemine on Anxiety-like Behaviors Induced by Chronic Unpredictable Mild Stress in Mice
Source: Brain Sci. 2022 Jan 30;12(2):191. doi: 10.3390/brainsci12020191 (PMC8870043; doi:10.3390/brainsci12020191)
Supplement: Supplementary file 1 [file brainsci-12-00191-s001.zip › brainsci-1569069-supplementary.pdf]

Suppressive effects of gelsemine on anxiety-like behaviors induced by chronic unpredictable mild stress in mice

Hui Yu[1,2], Mo-Huan Tang[1,2], Zi-Yue Zeng[1,2], Si-Juan Huang[1,2], Xiaofeng Zheng[1*],Zhao-Ying Liu[2*]

[1] *College of Veterinary Medicine, Hunan Agricultural University, Changsha, 410128, China*

[2]*Hunan Engineering Technology Research Center of Veterinary Drugs, Hunan Agricultural University, Changsha, 410128, China*

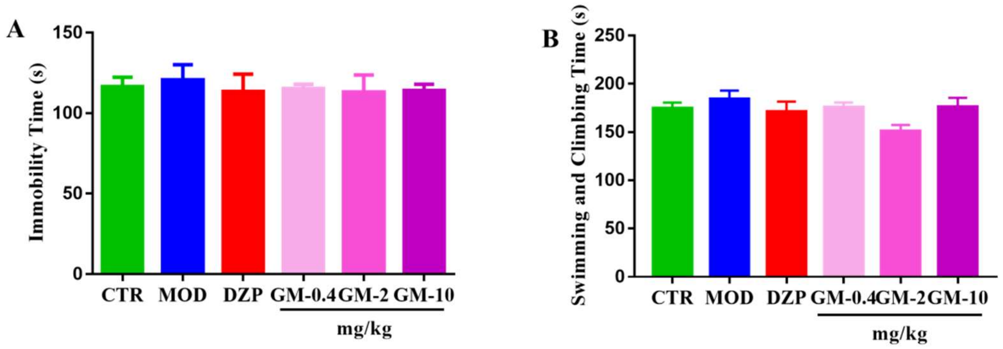

Figure S1. The immobility time and swimming and climbing time were measured in FST on day 27 (A-B, respectively). Mice were exposed to stress (subjected to CUMS) and daily treated with saline, gelsemine (GM, 0.4, 2.0 and 10.0 mg/kg, i.p.), diazepam (DZP, 1.0 mg/kg, i.p.) before test for 9 days. Data are presented as means the ± SEM （n=12）.

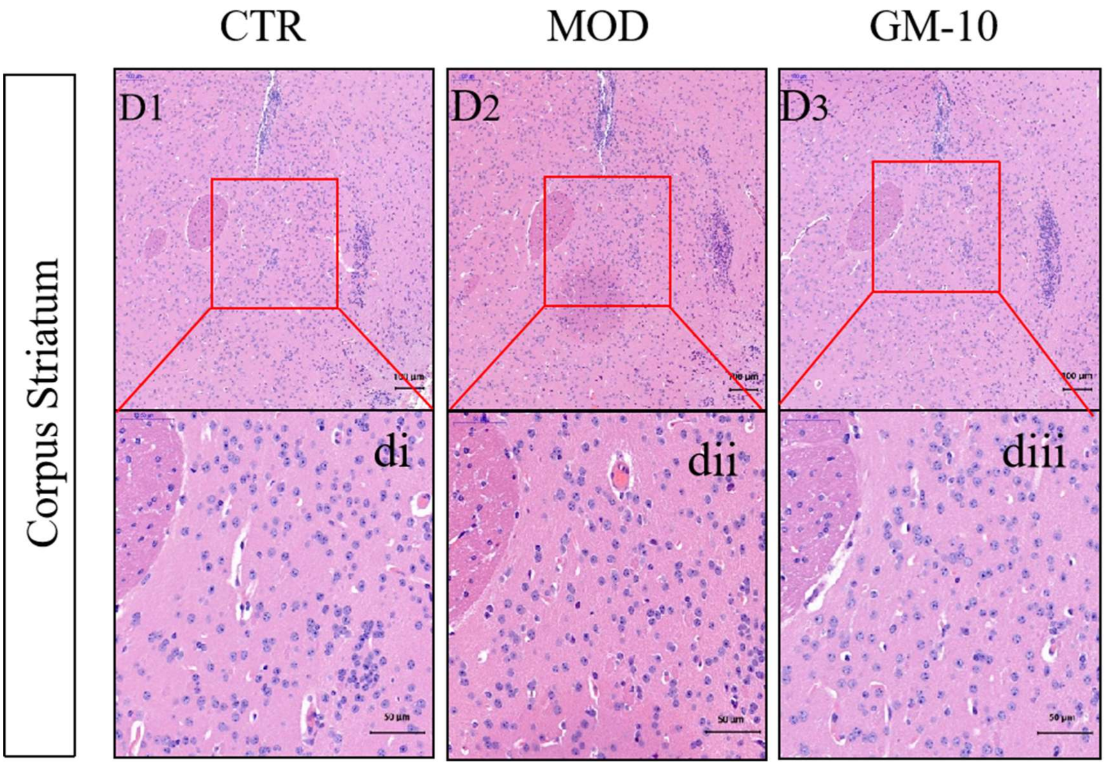

Figure S2. Effect of Gelsemine on the pathomorphology of Corpus striatum in mice. D1 represent the control group, D2 represent the model group, D3 represent the 10mg/kg Gelsemine - treated group, respectively (×100 magnification); di-diii represent the same group with ×400 magnification.
